# Mapping Forest Fuels through Vegetation Phenology: The Role of Coarse-Resolution Satellite Time-Series

**DOI:** 10.1371/journal.pone.0119811

**Published:** 2015-03-30

**Authors:** Sofia Bajocco, Eleni Dragoz, Ioannis Gitas, Daniela Smiraglia, Luca Salvati, Carlo Ricotta

**Affiliations:** 1 Consiglio per la Ricerca in Agricoltura e L’analisi Dell’Economia Agraria, Research Unit for Climatology and Meteorology applied to Agriculture (CRA-CMA), Rome, Italy; 2 Aristotle University of Thessaloniki, School of Forestry and Natural Environment, Thessaloniki, Greece; 3 Consiglio per la Ricerca in Agricoltura e l’Analisi Dell’Economia, Research Centre for Soil-Plant System studies (CRA-RPS), Rome, Italy; 4 University of Roma La Sapienza, Department of Environmental Biology, Rome, Italy

## Abstract

Traditionally fuel maps are built in terms of ‘fuel types’, thus considering the structural characteristics of vegetation only. The aim of this work is to derive a phenological fuel map based on the functional attributes of coarse-scale vegetation phenology, such as seasonality and productivity. MODIS NDVI 250m images of Sardinia (Italy), a large Mediterranean island with high frequency of fire incidence, were acquired for the period 2000–2012 to construct a mean annual NDVI profile of the vegetation at the pixel-level. Next, the following procedure was used to develop the phenological fuel map: (i) image segmentation on the Fourier components of the NDVI profiles to identify phenologically homogeneous landscape units, (ii) cluster analysis of the phenological units and post-hoc analysis of the fire-proneness of the phenological fuel classes (PFCs) obtained, (iii) environmental characterization (in terms of land cover and climate) of the PFCs. Our results showed the ability of coarse-resolution satellite time-series to characterize the fire-proneness of Sardinia with an adequate level of accuracy. The remotely sensed phenological framework presented may represent a suitable basis for the development of fire distribution prediction models, coarse-scale fuel maps and for various biogeographic studies.

## Introduction

Phenological studies of vegetation can be carried out at the species level (bud break, flowering, leaf flush, etc.) using in-situ field techniques [1]. However, only remote sensing can offer information on ecosystem phenology and productivity over several temporal scales and continuous spatial coverage. Space-borne optical sensors such as Moderate Resolution Imaging Spectroradiometer (MODIS) provide daily measurements of a variety of biophysical parameters of the land surface [2]. MODIS is a key instrument aboard the Terra and Aqua NASA satellites viewing the entire Earth's surface every 1 to 2 days, acquiring data in 36 spectral bands (http://modis.gsfc.nasa.gov/about/). These earth observation systems examine coarse-scale phenomena that allow retrievals of whole-system phenological metrics, such as the timing and magnitudes of greening, peak activity, and drying phases of the growing season [1]. At this landscape scale, the spatio-temporal dynamics of the vegetated land surface is often referred to as ‘land surface phenology’, as the remotely sensed phenology deals with mixtures of land cover and vegetation [3]. Land surface phenology thus provides information on key aspects of vegetation functionality, such as seasonality, productivity and inter-annual variability. Also, as vegetation functioning responds faster to environmental change and variability than vegetation structure and composition, there is a high potential for characterizing, classifying and mapping vegetation based on remotely sensed phenology [4–6].

Although, a number of studies have used remotely-sensed indices, such as the Normalized Difference Vegetation Index (NDVI), for the monitoring of vegetation dynamics from regional to global scales [7–9], the capability of remote sensing indicators to capture ecosystem functional dynamics has been only partially faced so far. The NDVI has been usually considered a reliable indicator of plant biomass and vegetation primary productivity [10–12]. Therefore, NDVI time series have been routinely used to measure vegetation dynamics and ecosystem phenology over large geographic areas [13–15]. Remote sensing derived vegetation phenology may provide an integrated measure of ecosystem responses to climatic factors, such as temperature and rainfall, as well as a synthetic quantification of the determinant conditions for fires and other disturbances [6].

Phenological classification characterizes and stratifies the land surface based on similar phenological patterns. White et al. [16] developed a global pheno-region database as a geographic framework for studying global climate change. They used Advanced Very High Resolution Radiometer (AVHRR) NDVI time series (8 km of pixel resolution) from 1982 to 1999 in conjunction with an eight-element monthly global climatology to generate global pheno-regions with a minimized probability of non-climatic forcing. Hargrove et al. [17] derived 15 phenological ecoregions based on clustering the similarities in five years (2002–2006) of cumulative MODIS NDVI data (250 m of pixel resolution). Gu et al. [18] performed a phenological classification of the conterminous United States based on MODIS NDVI time-series and a digital elevation model. The resulting pheno-class map is composed of 40 pheno-classes, each having unique phenological and topographic characteristics. Clerici et al. [19] explored the use of MODIS NDVI-derived phenology metrics for the identification and classification of Forest General Habitat Categories in Europe. Finally, De Angelis et al. [20] also used MODIS NDVI-derived phenology metrics to classify the vegetation of Sardinia (Italy) into clusters showing uniform fire behavior.

In the framework of fire risk assessment, variations in satellite-based NDVI values proved to be indicative of variations in water and nutrient availability, plant disease, and other stress factors, which are in turn indicators of a marked vulnerability of the vegetation to fire [21]. For this reason, over the years, many authors have proposed the use of multitemporal NDVI profiles to assess the proneness of vegetation to fire (for a detailed review, see [22]). Accordingly, as vegetation phenological status represents the main driver affecting fuel availability and moisture content, any investigation on fire behavior over large areas requires the ability of capturing spatio-temporal changes in vegetation phenology [20, 23].

Fire managers have tried to summarize the physical parameters and spatial distribution of fuel in different fuel types or fuel models [24–25]. More specifically, a fuel model has been defined as “an identifiable association of fuel elements of distinctive species, form, size, arrangement, and continuity that will exhibit characteristic fire behavior under defined burning conditions” [26]. Two well-known fire behavior fuel type systems are the Northern Forest Fire Laboratory (NFFL) system and the Canadian Forest Fire Behavior Prediction (FBP) system [27]. Also european researchers developed a new system, in the framework of the Prometheus project, which is better adapted to fuels found in Mediterranean ecosystems. These fuel types were defined for surface fire modelling, taking into account fuel height and density. However, traditional fuel maps, being built in terms of fuel types, represent the static, structural properties of fuel [28], not involving its dynamic, functional characteristics, such as moisture content, or vegetation health status.

In the wake of the work of De Angelis et al. [20], the aim of this paper is to use MODIS NDVI time-series (2000–2012) to develop a phenological fuel map of Sardinia (Italy) that takes into account the functional (rather than the structural) aspects of fuels, and to demonstrate its potential in characterizing fire risk (i.e. the chance of a fire to start).

## Study Area

Sardinia is an island located in the Mediterranean Sea between 38° 51' N and 41° 15' N latitude and 8° 8' E and 9° 50' E longitude, covering roughly 24000 km^2^. The island is characterized by a complex physical geography, with a prevalently hilly topography. The highest elevation is 1834 m, average elevation is 338 m. The island has a Mediterranean climate along the coast and a more continental climate on the interior mountain ranges. The average temperature is between 11 and 17° C, rainfalls have generally a Mediterranean distribution, with dry summers and two peaks of rainfall in spring and autumn. Mean annual precipitation is about 800 mm.

Land use along the coast and the main river valleys is dominated by agriculture that covers about 45% of the study area. Most urban areas of Sardinia are located in the coastal zone. In the interior areas, forest stands combined with pastures and shrublands prevail. The principal formations include *Quercus ilex* and *Quercus suber* forests. At higher elevations the sclerophyllous oak forests merge with broadleaved forests of *Quercus pubescens* and *Castanea sativa*.

The island is considered a wildfire hotspot, with a mean of more than 2500 events per year [29–30]. Fires are mainly of limited size, more than 67% of the events are smaller than 1 ha. Although most of the recorded fires are human-caused rather than ‘natural’ in origin, fire is a process largely correlated with climate. Like in most Mediterranean regions, in Sardinia fire is strongly seasonal, with the majority of fires occurring during the hottest and driest portion of the year. More than 98% of fires occur between May and October with a peak of fires (54%) in July and August.

## Data

In this study, information on the remotely sensed phenology of the vegetation of Sardinia was extracted from the 16-day MODIS 250 m NDVI maximum value composite product (MOD13Q1; https://lpdaac.usgs.gov/products/modis_products_table/mod13q1). Twenty-three NDVI images per year from 2000 to 2012 were acquired, for a total of 299 MODIS images. Late fall and winter scenes revealed several low quality and noisy pixels regions due to bad weather events. Therefore, we focused only on late spring-early autumn scenes, i.e. eleven yearly MODIS NDVI images (143 images in total) from Julian day 113 (April 23^rd^) to Julian day 273 (October 1^st^), which correspond to the months of highest fire occurrence. By averaging the NDVI values of each 16-day composite over the period 2000–2012, for each image pixel we derived a mean NDVI temporal profile showing mean seasonal variations in the NDVI values Wildfire data were provided by the Forest Service of Sardinia and contain all fires recorded from 2000 to 2010, together with the geographic (UTM) coordinates of their ignition points, and is assumed to be complete and reliable down to the smallest fires. The database used in this study includes all the fires recorded from April 23^rd^ to October 1^st^ during the period 2000–2010, and contains 28744 events.

## Methods

The following procedure was used to develop the phenological fuel map: (i) image segmentation on the Fourier components of the NDVI profiles to identify phenologically homogeneous landscape units, (ii) cluster analysis of the phenological units and post-hoc analysis of the fire-proneness of the phenological fuel classes (PFCs) obtained, (iii) environmental characterization (in terms of land cover and climate) of the PFCs.

### Fourier analysis and image segmentation

The vegetation (i.e. fuel) seasonality can only be captured by repeated measurements over time. Serial correlation among successive observations taken over a period of time reduces the statistical utility of multitemporal imagery [31]. Therefore, data reduction (usually ordination methods, like Principal Component Analysis) is typically applied to remove image correlation. However, in the ordination process, explicit reference to vegetation seasonality is lost, such that data reduction is achieved at the expense of ecological relevance. Alternative methods for retaining information on vegetation seasonality include polynomial functions [32] and Temporal Fourier Analysis (TFA) [23, 33–34]. Therefore, to analyze remotely-sensed fuel seasonality, we applied Temporal Fourier Analysis to the average NDVI profiles of each pixel.

TFA allows a complex signal to be expressed as the sum of a series of sine and cosine waves (harmonics) and an additive term [33]. The constant additive term corresponds to the arithmetic mean of the time series, while the harmonics correspond to the amplitudes into which the temporal signal is decomposed. TFA transforms a series of N observations taken at regular time intervals into a spectrum of (uncorrelated) harmonics at frequencies of 1, 1/2, 1/3, …, 2/N times the duration over which the N observations were made [31]. The obtained spectrum is a true partition of the variance of the series among frequencies, therefore spectral analysis, like TFA, represents a type of variance decomposition analysis [35]. In TFA there are as many harmonic variables as the data points. However, in practice only a few harmonics usually contribute substantially to the overall variance, and only these need to be calculated for capturing the main features of the vegetation seasonality [23, 31].

In this paper, we used late spring-early autumn NDVI profiles as our basic unit for TFA, the amplitude at one cycle period A_1_ (i.e. the first Fourier component) thus corresponds to the maximum variability of the NDVI values over the whole fire season, while the second Fourier component A_2_ summarizes the NDVI variability within the half-period of the NDVI profile. Accordingly, significant amplitude values associated to the first and second periodic terms indicate major variability of the NDVI profiles in response to the seasonal patterns of bioclimatic conditions. Finally, the additive term A_0_ is related to the summation of the NDVI values over the whole temporal profile [36] and can therefore be considered as a good indicator of the coarse-scale net primary productivity of vegetation on a seasonal basis. For additional details on Fourier analysis, see e.g. [37].

We used the TFA module of IDRISI Selva (Clark Labs, Worchester, MA) to extract the constant additive term (A_0_) and the amplitude values for the first and second Fourier harmonics (A_1_ and A_2_) of the seasonal NDVI profiles of each pixel, with 11 as number of time series images and 16 days as time lag between images. The constant and the first two amplitude output images of the TFA were then used for segmenting the territory of Sardinia into phenologically homogeneous units in the multispectral Fourier feature space.

**Fig 1:**
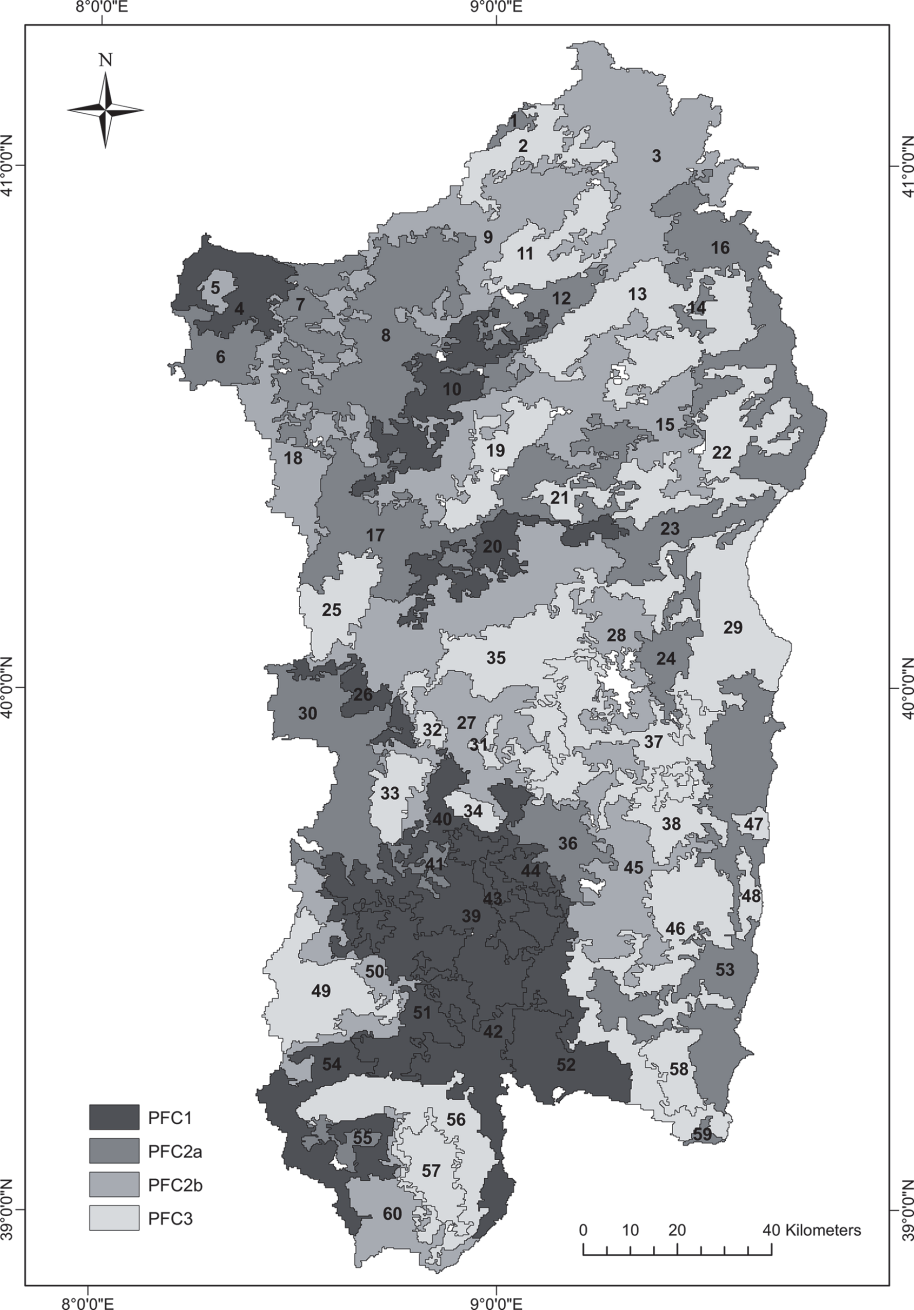
Spatial distribution of the 60 phenological units (PUs) and of the four phenological fuel classes (PFCs).

Image segmentation was conducted using the Fractal Net Evolution Approach [38] of the Definiens eCognition software (Definiens Imaging, Germany). In this bottom-up segmentation technique, individual pixels are perceived as the initial regions, which are sequentially merged pairwise into larger ones, with the intent of minimizing the heterogeneity of the resulting objects. The sequence of the merging objects, as well as the size and shape of the resulting objects, are empirically determined by the analyst [39]. A crucial unitless threshold, named scale parameter, is determined to specify the maximum allowed increase in the heterogeneity after a pairwise merge of the objects [40]. For the segmentation of the three TFA components two criteria were used: color (i.e. the value of each Fourier component for each pixel) and shape. The selection of the best value for each segmentation criterion was based on trial-and-error and on the visual interpretation of the results. Scale parameter is considered as the most effective parameter that is used to control the average image object size [41–42]. It depends on spatial resolution of the corresponding image and features of the study area. The higher value is selected for scale parameter, the larger the object is obtained. On these basis, the following values were selected for all three TFA components: scale parameter = 500, shape criterion = 0.2, and compactness = 0.8. The segmentation produced 60 phenological units (PUs) of at least 25 pixels in size, homogeneous in terms of mean seasonal NDVI timing (Fig. 1).

To quantify the fire-proneness of each PUs, the coordinates of the ignition points of all fires that occurred in the period 2000–2010 were overlaid on the PUs and the number of fires within each PU was computed. Next, we calculated a summary statistic of fire risk as the proportion of fires in each PU divided by the relative area of that PU in the study site [20]. This ratio, named ‘selection ratio’ (σ) [43, 29], takes values in the range [0, ∞]. If all PUs were equally fire prone, fire ignitions would occur randomly across the landscape, such that the number of ignitions within a given PU would be nearly proportional to the relative area of that PU and σ ≈ 1. Values larger than 1 denote PUs where ignitions occur more frequently than expected by chance alone, values lower than 1 denote PUs where ignition is less frequent than expected [29].

### Cluster analysis

In order to identify groups of phenological units with similar seasonality, we performed a hierarchical cluster analysis on the mean values of the Fourier components of each PU. Clustering is an agglomerative classification method for determining the intrinsic grouping in a set of data and identifying subpopulations of related objects with similar behavior with respect to some user-defined ecological property [44]. The cluster analysis was performed within the statistical software package Past 3.04 (free downloadable from http://folk.uio.no/ohammer/past/) using the Euclidean distance as similarity measure and the UPGMA method (average linkage between groups) as linkage criterion (see [35]). This analysis produced a dendrogram in which three distinctive phenological fuel classes (PFCs) homogeneous in terms of phenological patterns were detected: PFC1, PFC2 and PFC3. Based on our territorial knowledge, we divided the largest cluster into two subgroups: PFC2a (mainly distributed in the coastal zones) and PFC2b (mainly located in the inner parts).

Next, to evaluate the pyrological uniqueness of the four PFCs, we performed a permutational analysis of variance on the selection ratios of all PUs. Like for traditional univariate ANOVA, we calculated an F-value by dividing the between-group variability of the selection ratios by the corresponding within-group variability. A p‐value is then computed by permutation of the PUs among the different phenological classes (9999 randomizations, one-tailed test, see [45]).

Finally, in order to test the fire-proneness of the four PFCs, we first calculated the selection ratio of each class as the ratio between the relative proportion of fires in each class and the relative area of that class in the study site. Next, to determine whether the number of fires in each class is significantly different from random, we constructed the following Monte Carlo simulation. The 28744 fires that occurred in Sardinia during 2000–2010 were randomly reassigned to the PFCs, such that the probability of assignment of each fire to a given PFC was kept equal to its relative area. The null hypothesis is that fires occur randomly across the landscape, such that there is no difference between the relative abundance of fires in each class and its relative area within the analyzed landscape. Based on this procedure, we compared the actual value of σ for each PFC with the results of 9999 randomizations using a Microsoft Excel macro developed by Pezzatti et al. [46]. For each class, p-values (two-tailed test) were computed as the proportion of random values that were as low or lower (as high or higher) than the actual values.

### Environmental characterization of the phenological classes

To frame the PFCs within the environmental context of the study area, we analyzed the degree of association of each class with the land cover types and the climatic zonation of Sardinia. The land cover map was derived from the CORINE Land Cover (CLC) 2006 data [47]. The CLC data were aggregated into seven macro-classes: urban areas, arable lands, permanent crops, heterogeneous agricultural areas, forests, shrublands, and natural grasslands and pastures. Non-combustible classes, such as bare soils, wetlands and water bodies were excluded from further analysis. Because of the relative homogeneity of the CLC (macro-)classes in terms of fuel load and continuity, these classes were considered adequate for coarse-scale analysis of fire risk (see [29]).

The climatic zonation of the Island was obtained from the climatic map of Sardinia at scale 1:250,000 in Bajocco et al. [48]. According to this map, Sardinia is classified into three main climatic regions (CRs): Mediterranean, Transitional Mediterranean, and Transitional Temperate that are mainly characterized by a clear gradient of decreasing summer drought and mean annual temperatures.

To analyze the association of fuel phenology with land cover we first constructed a two-way contingency table in which the PFCs of all fire ignition points were associated to the corresponding CLC types (see [48]). Next, the actual values of all entries of the contingency table were compared to a distribution of 9999 random values (two-tailed test) obtained under the null hypothesis of no association between the phenological fuel classes and the land cover types. For all ignition points the association between the PFCs and the CLC types was randomized keeping constant the number of fires in each phenological and land use class. In addition, we also calculated a χ^2^ test statistic for the entire contingency table, the significance of which was tested with the same randomization procedure. The same approach was then used for analyzing the association between the phenological classes and the climatic regions of Sardinia.

## Results

**Fig 2:**
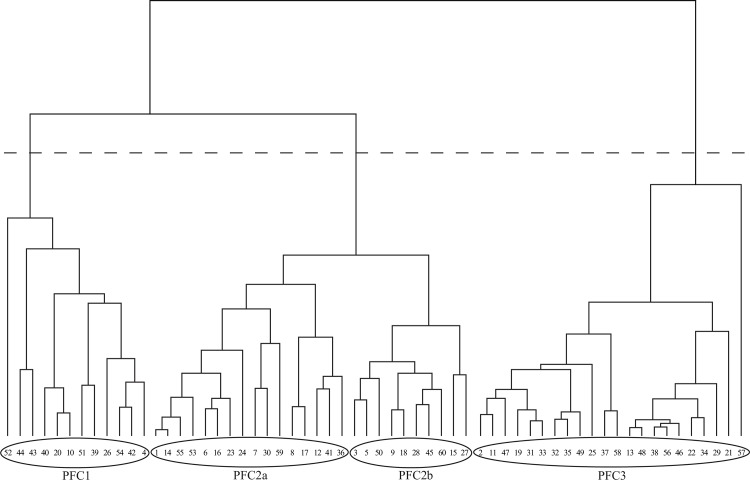
Dendrogram of the hierarchical cluster analysis of the mean values of the Fourier components for each phenological unit. The dashed line indicates the cutting level of the dendrogram. Circles indicate the grouping of the phenological units (PUs) into four clusters, named phenological fuel classes (PFCs), based on their phenological similarity in terms of vegetation seasonality and productivity.

Fig. 2 shows the dendrogram obtained from the cluster analysis of the phenological units of Sardinia (see also Fig. 1). Four clusters, PFC1 = (σ = 1.98), PFC2a (σ = 1.40), PFC2b (σ = 0.67) and PFC3 (σ = 0.32), were identified with selection ratios denoting decreasing fire risk from PFC1 to PFC3. According to the randomization test, fire incidence in terms of ignition is selective for all PFCs with very high significance (p < 0.001). The classes PFC1 and PFC2a showed high to moderately high fire-proneness, whereas PFC2b and PFC3 were characterized by significant fire-avoidance, meaning that the total number of fires observed in both classes is lower than expected by chance alone.

**Fig 3:**
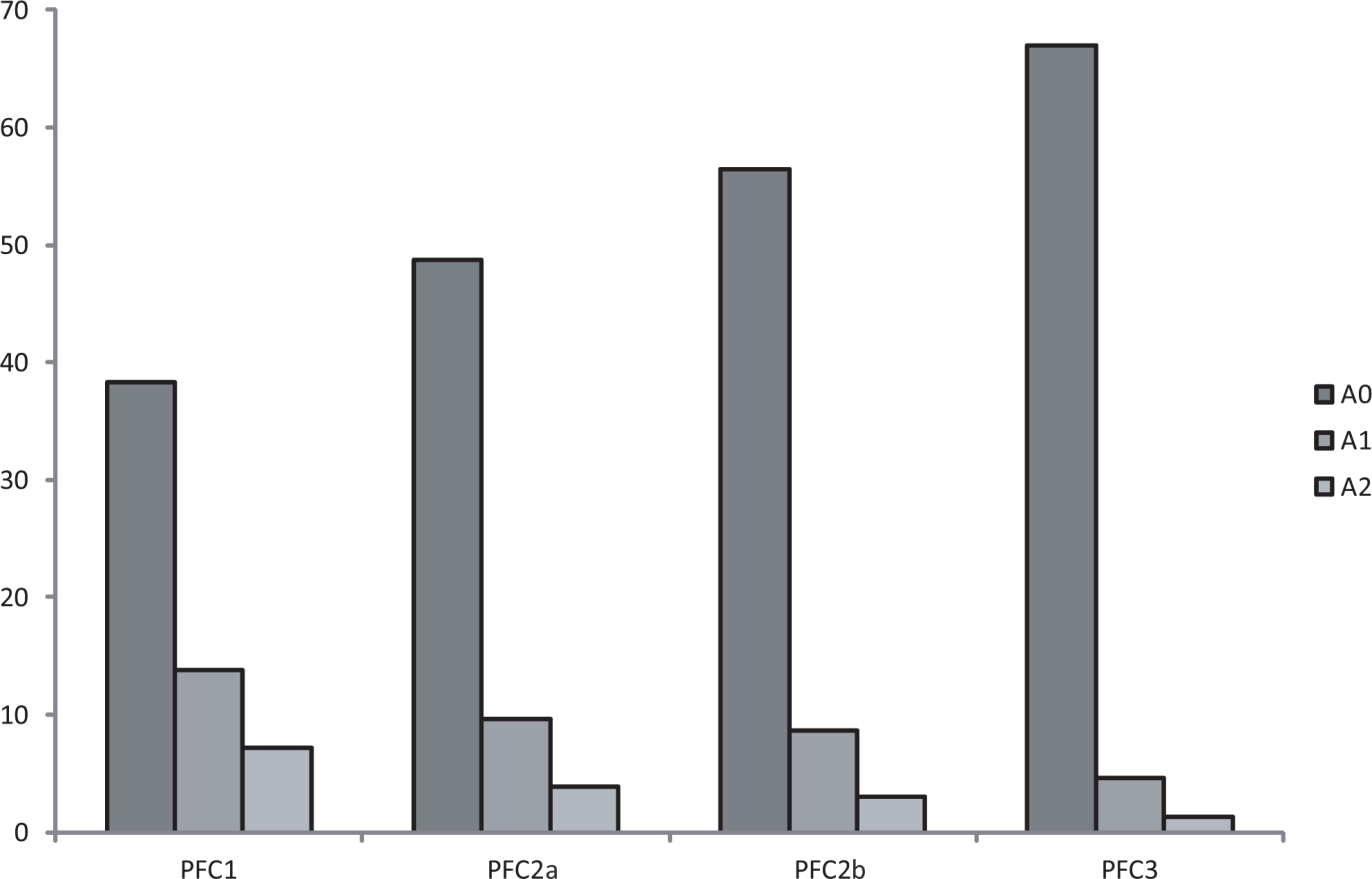
Graph of the mean values of each Fourier component for each phenological fuel class (PFC). The first (A1), and the second (A2) Fourier component represent the vegetation seasonality trend; the additive term (A0) expresses the vegetation productivity. The y-axis is in arbitrary units.

Fig. 3 shows the mean values of each Fourier component A_0_, A_1_ and A_2_ for each phenological fuel class. Results highlight that the additive term follows an increasing gradient from the highest to the lowest fire risk class, to the contrary, the values of the first and second harmonics progressively decrease from PFC1 to PFC3.

Overall, the permutational analysis of variance showed a significant pyrological difference between the PFCs (F = 30.56, p < 0.001). Likewise, the a-posteriori pairwise comparison between PFCs revealed that all classes are statistically unique in terms of fire-proneness at p < 0.05 (Table 1). The analysis of the degree of association between the phenological fuel classes and CLC types gave an observed value of χ^2^ = 9423.42 (p < 0.001). The same level of significance was found for the association between the PFCs and the CRs with an observed value of χ^2^ = 8097.30 (Table 2 and 3), denoting that the remotely sensed phenological fuel classes proved to be strongly associated both with the corresponding land cover types and the climatic regions of the study area.

**Table 1:** F-values of the a-posteriori pairwise comparisons between the phenological fuel classes (PFCs) identified by the cluster analysis.

PF classes	PFC1	PFC2a	PFC2b
**PFC2a**	7.841*	-	-
**PFC2b**	27.94	6.294*	-
**PFC3**	100.5	36.39	14.73

*significant at p = 0.05 (9999 randomizations, one-tailed test). The remaining values are all significant at p = 0.001. The value of the test statistic for the permutational ANOVA among all groups is F = 30.56 (p < 0.001).

**Table 2:** Contingency table showing the number of fires shared by the phenological fuel classes (PFCs) and the Corine Land Cover (CLC) types during the period 2000–2010.

		PF classes			
		PFC1	PFC2a	PFC2b	**PFC3**
**CLC types**	**Urban areas**	*852* ^**NS**^	*880*	353 ^NS^	110
	**Arable lands**	*5921*	3350	754	129
	**Permanent crops**	521	*760*	*216*	27
	**Heterogeneous agricultural areas**	2598	*3324*	*1699*	673
	**Forests**	176	242	*334*	**872**
	**Natural grasslands and pastures**	241	*808*	*386*	71
	**Shrublands**	381	977	*1186*	*903*

Italic characters indicate a positive association between PFC and land cover.

Normal characters indicate a negative association between PFC and land cover.

^NS^ indicates not significant at the p = 0.05 level (9999 randomizations, two-tailed test).

The remaining values are all significant at p = 0.001.

The value of χ^2^ for the whole contingency table is 9423.42 (p < 0.001).

**Table 3:** Contingency table showing the number of fires shared by the phenological fuel classes (PFCs) and the Climatic Regions (CRs) of Sardinia during the period 2000–2010.

		PF classes			
		PFC1	PFC2a	PFC2b	**PFC3**
**CRs**	**Mediterranean**	*8313*	*6093*	1290	561
	**Transitional Mediterranean**	0	317	*633*	*939*
	**Transitional Temperate**	2377	*3931*	*3005*	*1285*

Italic characters indicate a positive association between PFC and climate.

Normal characters indicate a negative association between PFC and climate.

All values are significant at p = 0.001 (9999 randomizations, two-tailed test).

The value of χ^2^ for the whole contingency table is 8097.30 (p < 0.001).

In particular, the class of highest fire risk, PFC1, shows a marked positive association with arable lands and a negative association with the natural and semi-natural land cover types. From a climatic viewpoint, PFC1 shows a significant positive association with the Mediterranean CR, and a negative association with both transitional CRs. The class of moderately high fire risk, PFC2a, is driven mainly by fuel size rather than by climate. Accordingly, PFC2a is positively associated to urban areas and to all land use types that are characterized by fine fuels, such as permanent crops and heterogeneous agricultural areas, which are usually located in the Mediterranean portion of the study area, and to natural grassland and pastures, which are mainly located in the Transitional Temperate region. At the same time, PFC2a is negatively associated to all coarse-fuel land use types, such as shrublands and forests.

To the contrary, the moderately low fire risk class, PFC2b, has a positive association with the Transitional Mediterranean and the Transitional Temperate CRs and a negative association with the Mediterranean CR. As concerns land use types, PFC2b is positively associated with all classes of low to very low human pressure, such as heterogeneous agricultural areas, pastures, shrublands and forests, which are mainly located at higher altitudes in the transitional CRs. Finally, PFC3 shows the same climatic preferences as PFC2b. However, unlike PFC2b, this latter class shows positive association only with shrublands and forests. The environmental characteristics of each PFC are summarized in Table 4.

**Table 4:** Summary of the environmental characteristics of the phenological fuel classes (PFCs) obtained from the analysis.

	Number of fires	Fire risk	Fuel type	Seasonal NDVI variability	Main Land Cover types	**Main Climatic types**
**PFC1 (4210.25 km** ^**2**^)	10690	High (σ = 1.98)	Fine fuel	Very high	Arable lands	Mediterranean
**PFC2a (5759.63 km** ^**2**^)	10341	Moderately high (σ = 1.40)	Fine fuel	High	Urban areas, Permanent crops, Heterogeneous agricultural areas and Natural grasslands and pastures	Mediterranean Transitional Temperate
**PFC2b (5743.31 km** ^**2**^)	4928	Moderately low (σ = 0.67)	Coarse fuel	Low	Permanent crops, Heterogeneous agricultural areas, Natural grasslands and pastures, Forest and Shrublands	Transitional Mediterranean Transitional Temperate
**PFC3 (6691.31 km** ^**2**^)	2785	Low (σ = 0.32)	Coarse fuel	Very low	Forest and Shrublands	Transitional Mediterranean Transitional Temperate

## Discussion

This paper follows the wake of the work of De Angelis et al. [20] and shares with it the working hypothesis and the methodological flow; the general objective of both studies, in fact, is to classify the vegetation by means of MODIS NDVI time-series. However, unlike De Angelis et al. [20], the specific aim of this paper is not to investigate the phenological metrics that best characterize the fire selectivity behavior, but to introduce a coarse-resolution satellite-based framework that can be reliably used for characterizing fire risk (i.e. the chance of a fire to start) and easily developed regardless the availability of complete wildfire database or specific fuel data.

Fire risk (or ignition risk) is defined as “the chance of a fire starting as determined by the presence and activity of any causative agent” [49]. When dealing with fire fighting strategies, fire risk is the most important variable to consider. Knowing the fire ignitions patterns and their determinants may represent a decision support tool about where the intervention forces should be concentrated for time- and cost-efficient actions. Furthermore, unlike fire risk, the fire spreading probability is highly dependent on the presence of severe weather conditions, rather than on fuel characteristics alone [48, 50–52]. Therefore, focusing on fire ignition may allow to analyze the correlation between (remotely sensed) fuel conditions and fire occurrence without the confounding effects of extreme meteorological events [49].

Understanding the geographic distribution of vegetation phenology (i.e., the timing of fuel availability) may be key to planning effective fire fighting and prevention strategies, since regions with similar fuel phenology patterns, and hence with similar fire incidence, are regions where similar management policies should be applied [53]. According to our results, NDVI seasonal timing represents a main functional driver of fire risk, in terms of both fuel load and flammability. Hence, it can be adequately used to classify the territory into fuel classes with distinctive phenological and pyrological behaviour, which in turn reflects underlying differences in land cover and climate.

From a biological viewpoint, the discriminating factors among the four PFCs were represented by the vegetation seasonal productivity (i.e. the fuel amount cumulated during the observed season) and the seasonal variability of the NDVI values, which expresses the fuel flammability in terms of transition from moist to dry vegetation. The highest fire risk classes, PFC1 and PFC2a, were characterized by a low fuel amount during the fire season and a high variability in the NDVI values. To the contrary, the lowest fire risk categories, PFC2b and PFC3, were characterized by high seasonal fuel load and low variability in the NDVI timing.

Looking at land cover and climate, the classes with the highest fire risk are mainly associated to Mediterranean climates and to urban areas, agriculture and pastures. More specifically, while PFC1 is mainly associated with urban and agricultural areas, PFC2a is associated with a more heterogeneous vegetation cover, including grasslands and pastures in more temperate areas at higher altitudes. These land cover types are generally characterized by high human pressure and fine fuel that dries quickly and can therefore burn easily after short periods of dry weather [29]. In particular, in Mediterranean regions fire has been traditionally used as a land management tool for creating pastures or eliminating agricultural waste. On the other hand, the classes with the lowest fire risk are mainly located in areas with higher rainfall and mostly covered by woody vegetation. Such coarser forest fuels take longer to dry. Therefore, they can burn only after longer (and thus infrequent) periods of dry weather. While PFC2b is mainly covered by agricultural and more natural land cover types with different degrees of human impact, PFC3 is mainly associated with shrublands and forests, showing the highest fire-avoidance due to the coexistence of reduced human impact, coarser fuels and limited summer drought.

These findings are in agreement with previous studies on fire risk in the Mediterranean basin. For instance, Bajocco & Ricotta [29] and Guglietta et al. [54] showed that the highest probability of fire occurrence in Sardinia is associated with agricultural and urban areas, due to the heaviest human presence, which represents the main source of fire ignition. In contrast, the lowest fire ignition risk can be found in the inner and higher portions of the study region, where population density is much lower. In Portugal, Catry et al. [55] emphasized the role of agriculture in driving the spatial distribution of fire events, due to the fine fuel load and the land management practices (e.g. burning crop residues). Finally, Bajocco et al. [48] in Sardinia (Italy), Pausas & Fernández-Muñoz [51] in the province of Valencia (Spain) and Oliveira et al. [56] across Mediterranean Europe, all emphasized the combined effect of land cover (which determines the fuel amount) and climate (which determines the fuel-proneness to burn) on fire occurrence.

In conclusion, MODIS NDVI time-series may represent a suitable basis for the development of coarse-scale dynamic fuel maps and fire prediction models. In addition, remotely sensed phenological data may also have additional value for various wildfire-oriented applications related to land surface monitoring and assessment (see [57]), including drought monitoring, ecological modeling of the impact of global change on burning patterns [58–59], or the identification of main areas of pre-fire intervention [18]. Furthermore, the possibility of updating the remotely-sensed fuel maps year by year with low-cost, easy to retrieve, and homogeneous data allows to keep them current so that they will retain their value for users as landscapes change.

## References

[1] Ma X, Huete A, Yu Q, Restrepo Coupe N, Davies K, Broich M, et al (2013) Spatial patterns and temporal dynamics in savanna vegetation phenology across the North Australian Tropical Transect. Remote Sens Environ 139: 97–115.

[2] Friedl MA, McIver DK, Hodges JCF, Zhang XY, Muchoney D, Strahler AH, et al (2002) Global land cover mapping from MODIS: Algorithms and early results. Remote Sens Environ 83: 287–302.

[3] Brown ME, de Beurs K, Vrieling A (2010) The response of African land surface phenology to large scale climate oscillations. Remote Sens Environ 114: 2286–2296.

[4] Alcaraz D, Paruelo J, Cabello J (2006) Identification of current ecosystem functional types in the Iberian Peninsula. Global Ecol Biogeogr 15: 200–212.

[5] Paruelo JM, Golluscio RA, Guerschman JP, Cesa A, Jouve VV, Garbulsky MF (2004) Regional scale relationships between ecosystem structure and functioning: the case of the Patagonian steppes. Global Ecol Biogeogr 13: 385–395.

[6] Wessels K, Steenkamp K, von Maltitz G, Archibald S (2011) Remotely sensed vegetation phenology for describing and predicting the biomes of South Africa. Appl Veg Sci 14: 49–66.

[7] Fensholt R, Langanke T, Rasmussen K, Reenberg A, Prince SD, Tucker CJ, et al (2012) Greenness in semi-arid areas across the globe 1981–2007—an earth observing satellite based analysis of trends and drivers. Remote Sens Environ 121: 144–158.

[8] Ivits E, Cherlet M, Tóth G, Sommer S, Mehl W, Vogt J, Micale F (2012) Combining satellite derived phenology with climate data for climate change impact assessment. Global Planet Change 88–89: 85–97.

[9] Jeong SJ, Ho CH, Gim HJ, Brown ME (2011) Phenology shifts at start vs. end of growing season in temperate vegetation over the Northern Hemisphere for the period 1982–2008. Glob Change Biol 17: 2385–2399.

[10] Glenn EP, Huete AR, Nagler PL, Nelson SG (2008) Relationship between remotely-sensed vegetation indices, canopy attributes and plant physiological processes: What vegetation indices can and cannot tell us about the landscape. Sensors 8: 2136–2160. 27879814 10.3390/s8042136 PMC3673410

[11] Bajocco S, De Angelis A, Salvati L (2012) A satellite-based green index as a proxy for vegetation cover quality in a Mediterranean region. Ecol Indic 23: 578–587.

[12] Manes F, Ricotta C, Salvatori E, Bajocco S, Blasi C (2010) A multiscale analysis of canopy structure in Fagus sylvatica L. and Quercus cerris L. old-growth forests in the Cilento and Vallo di Diano National Park. Plant Biosyst 144: 202–210.

[13] Zhang XY, Friedl MA, Schaaf CB, Strahler AH, Hodges JCF, Gao F, et al (2003) Monitoring vegetation phenology using MODIS. Remote Sens Environ 84: 471–475.

[14] Ahl DE, Gower ST, Burrows SN, Shabanov NV, Myneni RB, Knyazikhin Y (2006) Monitoring spring canopy phenology of a deciduous broadleaf forest using MODIS. Remote Sens Environ 104: 88–95.

[15] Törmä M, Kervinen M, Anttila S (2011) Estimating vegetation phenological trends using MODIS NDVI time series. Proceedings of SPIE, Earth Resources and Environmental Remote Sensing/GIS Applications II 81810P, 14 pp.

[16] White MA, Hoffman FM, Hargrove WW, Nemani RR (2005) A global framework for monitoring phenological responses to climate change. Geophys Res Lett 32: L04705, 10.29/2004GL021961

[17] Hargrove WW, Spruce JP, Gasser GE, Hoffman FM (2009) Toward a national early warning system for forest disturbances using remotely sensed canopy phenology. Photogramm Eng Remote Sens 75: 1150–1156.

[18] Gu Y, Brown JF, Miura T, van Leeuwen WJD, Reed BC (2010) Phenological Classification of the United States: A Geographic Framework for Extending Multi-Sensor Time-Series Data. Remote Sens 2: 526–544.

[19] Clerici N, Weissteiner CJ, Gerard F (2012) Exploring the Use of MODIS NDVI-Based Phenology Indicators for Classifying Forest General Habitat Categories. Remote Sens 4: 1781–1803.

[20] De Angelis A, Bajocco S, Ricotta C (2012) Phenological variability drives the distribution of wildfires in Sardinia. Landscape Ecol 27: 1535–1545.

[21] Fiorucci P, Gaetani F, Lanorte A, Lasaponara R (2007) Dynamic Fire Danger Mapping from Satellite Imagery and Meteorological Forecast Data. Earth Interact 11: 1–17.

[22] Lasaponara R (2005) Inter-comparison of AHVRR-based fire susceptibility indicators for the Mediterranean ecosystems of Southern Italy. Int J Remote Sens 26: 853–870.

[23] Bajocco S, Rosati L, Ricotta C (2010a) Knowing fire incidence through fuel phenology: A remotely sensed approach. Ecol Model 221: 59–66.

[24] Anderson HE (1982) Aids to determining fuels models for estimating fire behavior. General Technical Report INT-122, Intermountain Forest and Range Experiment Station, USDA Forest Service, Ogden, 22 pp.

[25] Burgan R, Rothermel RC (1984) BEHAVE: Fire behavior prediction and fuel modeling system-FUEL subsystem. General Technical Report INT-167, USDA Forest Service, Washington, 126 pp.

[26] Merrill DF, Alexander ME (1987) Glossary of Forest Fire Management Terms. National Research Council of Canada, Committee for Forest Fire Management, 91 pp.

[27] Arroyo LA, Pascual C, Manzanera JA (2008) Fire models and methods to map fuel types: The role of remote sensing. Forest Ecol Manag 256: 1239–1252.

[28] Lanorte A, Lasaponara R (2008) Fuel type characterization based on coarse resolution MODIS satellite data. iForest 1: 60–64.

[29] Bajocco S, Ricotta C (2008) Evidence of selective burning in Sardinia (Italy): Which land-cover classes do wildfires prefer? Landscape Ecol 23: 241–248.

[30] Bajocco S, Salvati L, Ricotta C (2011) Land degradation versus fire: A spiral process? Prog Phys Geogr 35: 3–18.

[31] Scharlemann JP, Benz D, Hay SI, Purse BV, Tatem AJ, Wint GR, Rogers DJ (2008) Global data for ecology and epidemiology: a novel algorithm for temporal Fourier processing MODIS data. PloS one 3: e1408 10.1371/journal.pone.0001408 18183289 PMC2171368

[32] Jönsson P, Eklundh L (2002) Seasonality extraction by function fitting to time-series of satellite sensor data. IEEE Trans. Geosci Remote Sens 40: 1824–1832.

[33] Jakubauskas ME, Legates DR, Kastens JH (2002) Crop identification using harmonic analysis of time-series AVHRR NDVI data. Comput Electron Agr 37: 127–139.

[34] Julien Y, Sobrino JA, Verhoef W (2006) Changes in land surface temperatures and NDVI values over Europe between 1982 and 1999. Remote Sens Environ 103: 43–55.

[35] Legendre P, Legendre L (1998) Numerical Ecology. Amsterdam: Elsevier, pp. 673–682.

[36] Ricotta C, Avena G, De Palma A (1999) Mapping and monitoring net primary productivity with AVHRR NDVI time-series: Statistical equivalence of cumulative vegetation indices. ISPRS J. Photogramm. Remote Sens 54: 325–331.

[37] Azzali S, Menenti M (2000) Mapping vegetation–soil–climate complexes in southern Africa using temporal Fourier analysis of NOAA-AVHRR NDVI data. Int J Remote Sens 21: 973–996.

[38] Baatz M, Schäpe A (2000) Multiresolution Segmentation: an optimization approach for high quality multi-scale image segmentation, In: Strobl J., Blaschke T., Griesebner G. (Eds.), Angewandte Geographische Informationsverarbeitung XII, Beiträge zum AGIT-Symposium Salzburg, Wichmann, Heidelberg, pp. 12–23.

[39] Mallinis G, Koutsias N, Tsakiri-Strati M, Karteris M (2008) Object-based classification using Quickbird imagery for delineating forest vegetation polygons in a Mediterranean test site. ISPRS J Photogramm Remote Sens 63: 237–250.

[40] Benz UC, Hofmann P, Willhauck G, Lingenfelder I, Heynen M (2004) Multiresolution, object-oriented fuzzy analysis of remote sensing data for GIS-ready information. ISPRS J Photogramm Remote Sens 58: 239–258.

[41] Li C, Shao G (2012) Object-oriented classification of land use/cover using digital aerial orthophotography. Int J Remote Sens 33: 922–938.

[42] Lowe SH, Guo X (2011) Detecting an Optimal Scale Parameter in Object-Oriented Classification. IEEE J Sel Topics Appl Earth Observ 4: 890–895.

[43] Moreira F, Rego FC, Ferreira PG (2001) Temporal (1958–1995) pattern of change in a cultural landscape of northwestern Portugal: implications for fire occurrence. Landscape Ecol 16: 557–567.

[44] Pollard KS, van der Laan MJ (2008) Supervised distance matrices. Stat Appl Genet Mol 7: Article 33. 10.2202/1544-6115.1404 19049489

[45] Anderson MJ (2001) A new method for non-parametric multivariate analysis of variance. Austral Ecol 26: 32–46. 11469182

[46] Pezzatti GB, Bajocco S, Torriani D, Conedera M (2009). Selective burning of forest vegetation in Canton Ticino (southern Switzerland). Plant Biosyst 143, 609–620.

[47] EEA (2007) CLC2006 technical guidelines. Technical report 17/2007. Copenhagen: European Environment Agency, pp 66.

[48] Bajocco S, Pezzatti GB, Mazzoleni S, Ricotta C (2010b) Wildfire seasonality and land use: When do wildfires prefer to burn? Environ Monit Assess 164: 445–452. 10.1007/s10661-009-0905-x 19396557

[49] Conedera M, Torriani D, Neff C, Ricotta C, Bajocco S, Pezzatti GB (2011) Using Monte Carlo simulations to estimate relative fire ignition danger in a low-to-medium fire-prone region. For Ecol Manag 261: 2179–2187.

[50] Nunes MCS, Vasconcelos MJ, Pereira JMC, Dasgupta N, Alldredge RJ, Rego FC (2005) Land Cover Type and Fire in Portugal: Do Fires Burn Land Cover Selectively? Landscape Ecol 20: 661–673.

[51] Pausas J G, Fernández-Muñoz S (2012) Fire regime changes in the Western Mediterranean Basin: from fuel-limited to drought-driven fire regime. Clim Chang 110: 215–226. 10.1007/s11060-012-0954-9 22890970

[52] Pereira MG, Trigo RM, da Camara CC, Pereira JMC, Leite SM (2005) Synoptic patterns associated with large summer forest fires in Portugal. Agr Forest Meteorol 129: 11–25.

[53] Curt T, Borgniet L, Bouillon C. (2013) Wildfire frequency varies with the size and shape of fuel types in southeastern France: Implications for environmental management. J Environ Manag 117: 150–161. 10.1016/j.jenvman.2012.12.006 23369835

[54] Guglietta D, Conedera M, Mazzoleni S, Ricotta C (2011) Mapping fire ignition risk in a complex anthropogenic landscape. Remote Sens Lett 2: 213–219.

[55] Catry FX, Rego FC, Bação F (2009) Modeling and mapping wildfire ignition risk in Portugal. Int J Wildland Fire 18: 921–931.

[56] Oliveira S, Oehler F, San-Miguel-Ayanz J, Camia A, Pereira JMC (2012) Modeling spatial patterns of fire occurrence in Mediterranean Europe using multiple regression and random forest. For Ecol Manag 275: 117–129.

[57] Nemani R, Hashimoto H, Votava P (2009) Monitoring and forecasting ecosystem dynamics using the Terrestrial Observation and Prediction System (TOPS). Remote Sens Environ 113: 1497–1509.

[58] Morisette JT, Richardson AD, Knapp AK (2009) Tracking the rhythm of the seasons in the face of global change: phenological research in the 21st century. Front Ecol Environ 7: 253–260.

[59] Thonicke K, Cramer W (2006) Long-term trends in vegetation dynamics and forest fire in Brandenburg (Germany) under a changing climate. Nat Hazards 38: 283–300.

