# Correction: Mapping Forest Fuels through Vegetation Phenology: The Role of Coarse-Resolution Satellite Time-Series

**DOI:** 10.1371/journal.pone.0126430

**Published:** 2015-04-13

**Authors:** The PLOS ONE Staff

The second author’s name is spelled incorrectly. The correct name is: Eleni Dragozi. The correct citation is: Bajocco S, Dragozi E, Gitas I, Smiraglia D, Salvati L, Ricotta C (2015) Mapping Forest Fuels through Vegetation Phenology: The Role of Coarse-Resolution Satellite Time-Series. PLoS ONE 10(3): e0119811. doi: 10.1371/journal.pone.0119811

The publisher apologizes for the error.

## References

[1] Bajocco S, Dragoz E, Gitas I, Smiraglia D, Salvati L, Ricotta C (2015) Mapping Forest Fuels through Vegetation Phenology: The Role of Coarse-Resolution Satellite Time-Series. PLoS ONE 10(3): e0119811 doi: 10.1371/journal.pone.0119811 25822505 10.1371/journal.pone.0119811 PMC4379084

